# Validation of 3D documentation of palatal soft tissue shape, color, and irregularity with intraoral scanning

**DOI:** 10.1007/s00784-017-2198-8

**Published:** 2017-10-06

**Authors:** Julie T. Deferm, Ruud Schreurs, Frank Baan, Robin Bruggink, Matthijs A. W. Merkx, Tong Xi, Stefaan J. Bergé, Thomas J. J. Maal

**Affiliations:** 1 Department of Oral and Maxillofacial Surgery, Radboud University Nijmegen Medical Centre, P.O. Box 9101, 6500, HB Nijmegen, Postal number 590 the Netherlands; 2 Department of Oral and Maxillofacial Surgery, Academic Medical Centre of Amsterdam (AMC), Academic Centre for Dentistry (ACTA), Amsterdam, the Netherlands; 3 Department of Orthodontics and Craniofacial biology, Radboud University Nijmegen Medical Centre, Nijmegen, the Netherlands

**Keywords:** Intraoral scan, TRIOS® 3Shape, 3D Imaging, Oral oncology, Palatal soft tissue, Digital dental models

## Abstract

**Objectives:**

The purpose of this study was to assess the feasibility of 3D intraoral scanning for documentation of palatal soft tissue by evaluating the accuracy of shape, color, and curvature.

**Materials and methods:**

Intraoral scans of ten participants’ upper dentition and palate were acquired with the TRIOS® 3D intraoral scanner by two observers. Conventional impressions were taken and digitized as a gold standard. The resulting surface models were aligned using an Iterative Closest Point approach. The absolute distance measurements between the intraoral models and the digitized impression were used to quantify the trueness and precision of intraoral scanning. The mean color of the palatal soft tissue was extracted in HSV (hue, saturation, value) format to establish the color precision. Finally, the mean curvature of the surface models was calculated and used for surface irregularity.

**Results:**

Mean average distance error between the conventional impression models and the intraoral models was 0.02 ± 0.07 mm (*p* = 0.30). Mean interobserver color difference was − 0.08 ± 1.49° (*p* = 0.864), 0.28 ± 0.78% (*p* = 0.286), and 0.30 ± 1.14% (*p* = 0.426) for respectively hue, saturation, and value. The interobserver differences for overall and maximum surface irregularity were 0.01 ± 0.03 and 0.00 ± 0.05 mm.

**Conclusions:**

This study supports the hypothesis that the intraoral scan can perform a 3D documentation of palatal soft tissue in terms of shape, color, and curvature.

**Clinical relevance:**

An intraoral scanner can be an objective tool, adjunctive to the clinical examination of the palatal tissue.

## Introduction

A thorough clinical oral examination (COE) and follow up is essential in detecting possible intraoral pathology [1–4]. Oral soft-tissue lesions can be documented in many ways. Standard COE requires a meticulous head and neck examination, palpation, and visual inspection of the oral mucosa. In general, the clinician starts to describe the location of abnormal tissue, using anatomic landmarks or fixed reference points. The size of the lesion can be estimated or measured with a ruler or probe. Additional characteristics such as growth pattern, color changes, consistency of the mucosa, and mobility of the underlying tissues should be described [1, 4]. These clinical features should be documented in detail in the patient’s file to enable communication with other clinicians and to detect any changes or suspicious signs for malignancy during follow-up.

Early recognition and diagnosis of oral pathology is crucial since precancerous lesions can evolve in malignant tumors and survival rates are higher, and overall morbidity and mortality are lower if oral cancer is still localized [2]. COE is a subjective process, depending on the experience of the clinician. Assessing resolution or aggravation of an oral lesion is a problematic issue if different professionals perform follow-up [2, 3]. Adjunctive aids have been developed to enhance reproducible and objective documentation of soft tissue lesions. Digital two-dimensional photographs of suspected mucosal lesions for example are an essential addition to the clinical examination. Yet, acquisition of intraoral photographs in a reproducible fashion is extremely challenging. Differences in angulation, lighting, and magnification are all factors that hamper comparison of a lesion’s size and color between different photographs. Other screening aids for the detection of suspected oral lesions such as toluidine blue staining, tissue reflectance (ViziLite plus, MicroLux DL), and narrow-emission fluorescence (VELscope) can be helpful in early detection of malignancy [3, 4]. Unfortunately, no technique or technology to date has provided definitive evidence to suggest that it improves the detection of oral cancer beyond COE alone [4]. Innovative 3D imaging techniques, such as intraoral scanning (IOS), could create the possibility of a more complete analysis of the morphological characteristics of a suspected lesion, providing a better identification of (pre) malignant tumors.

The purpose of this study is to assess the feasibility of 3D intraoral scanning as an adjunctive tool for documentation of palatal soft tissue by evaluating the accuracy of shape, color, and curvature of the scans.

## Materials and methods

### Patients

This study was conducted at the Department of Oral and Maxillofacial Surgery at the Radboud University Medical Centre Nijmegen, the Netherlands, in accordance with the Declaration of Helsinki guidelines on medical human research ethics (2014–1484). Ten healthy Caucasian volunteers were recruited for this pilot study. Inclusion criteria were a minimum age of 18 years and fully permanent dentition (except third molars); exclusion criteria were known pathological oral lesions in the upper jaw, limitations in mouth opening, edentulous patients, or patients with active orthodontic treatment or severely decayed teeth. Informed consent was obtained from all individual participants included in the study.

### Data acquisition

Intraoral scans of the participants’ upper dentition and palate were acquired with the TRIOS® 3D intraoral scanner (POD TRIOS® 3, 3Shape™, Copenhagen, Denmark; software version: TRIOS 2015–1). All participants were scanned by two independent observers according to the scanning protocol provided by the manufacturer [5]. Observer 2 was experienced in the use of the intraoral scanner, whereas observer 1 had never used the intraoral scanner before. The scan time of both examiners was recorded. Impressions of the upper jaw were taken with an irreversible hydrocolloid material (ALG; Blue-print Cremix; Dentsply Int.) since this impression material is less resistant to possible deformation. A Cavex alginate mixer was used, and the impressions were immediately scanned with a laser-surface scanner (D500 3D scanner, 3Shape™, Copenhagen, Denmark), to produce a 3D laser surface model of the conventional impression (digitized conventional impression). The digitized conventional impression and the models acquired with the intraoral scanner were exported in a Wavefront object file format (OBJ).

### Trueness and precision palatal soft tissue

**Fig. 1:**
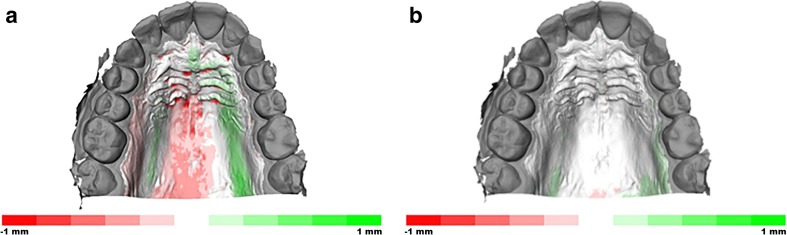
**a** Illustration of the color-coded distance map that visualizes the trueness of the palatal area between the plaster model and IOS. **b** Illustration of the color-coded distance map that visualizes the precision of the palatal area between the IOS of both observers

The palatal surface distal to the second molars was removed in 3DS Max (v2016, Autodesk Inc., San Rafael, CA, USA). The laser surface models of the conventional impression and of the intraoral scans of both observers were imported in Maxilim software (V2.3.0, Medicim NV, Mechelen, Belgium). An Iterative Closest Point (ICP) algorithm was used to align the IOS models on the upper dentition; this proved to be highly accurate in earlier publications [5–7]. The inter-surface distances between the palatal regions of the aligned models were calculated and visualized as a color-coded distance map (Fig. 1a, b). The absolute average distance and absolute 90th percentile distance was calculated for each volunteer as a measure of precision. Each intraoral scan model was separately aligned with the digitized conventional impression, and the absolute average and absolute 90th percentile distance was calculated for the models of each volunteer to assess trueness. The average of the distances (non-absolute) between digitized impression and intraoral scans was used as a measure of difference in model size.

### Color measurements

**Fig. 2:**
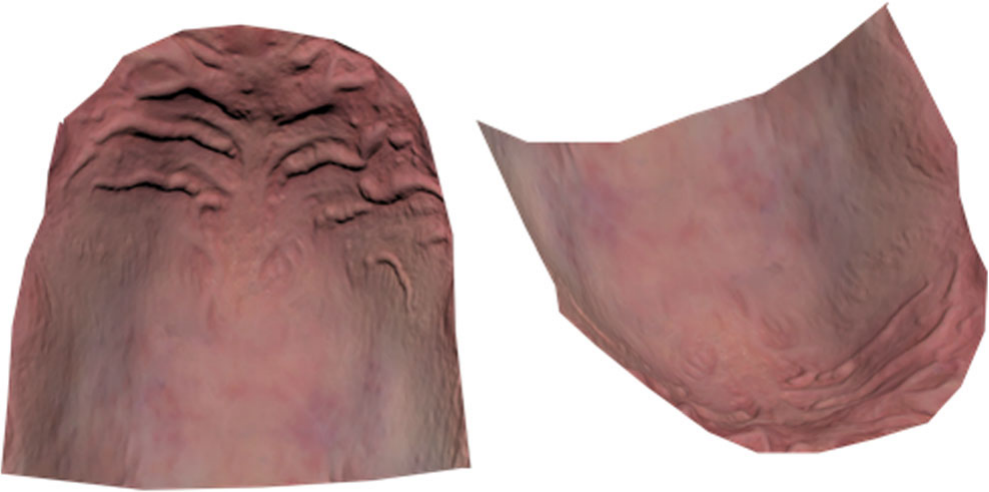
The palate after extraction from the intraoral scan. Different shades of red can be distinguished

To investigate the precision of the color measurements of the intraoral scanner, the palate was extracted from the intraoral scans (Fig. 2) in 3DS Max in HSV (hue, saturation, value) format. The HSV color scale is a cylindrical coordinate representation of the RGB color scale. Hue is represented as a value between 0 and 360 (degrees) and can be regarded as the basic feature of color. Saturation describes the purity of the color (0–100 scale) and value the brightness of the color (0–100 scale) [8]. The overall palatal color between both observers was calculated.

### Irregularity of the surface

The surface models of both IOS scans were subsequently imported in Matlab (v2015b, the Mathworks Inc., Natick, MA, USA). The mean curvature (*H*) was calculated for all vertices. Overall surface irregularity was assessed by calculating the mean of the absolute curvature values for each patient for both observers. The 90th percentile of the absolute curvatures was used as a measure of maximum surface irregularity of the palate.

### Statistical analysis

All data analysis was performed using IBM SPSS Statistics (version 22, IBM Corp., Armonk, NY). A paired *t* test was performed on the scan time data of the experienced and inexperienced observer, to evaluate the effect of training on scanning time. Descriptive statistics (mean, standard deviation) were calculated for the absolute average distance and absolute 90th percentile distance measure between the intraoral scans (precision) and for the absolute average distance and absolute 90th percentile distance between intraoral scans and digitized conventional impression (trueness). A one-sided *t* test of the (non-absolute) average distances between the intraoral scan model and digitized model was performed to evaluate whether the size of the models differed significantly (H0: no size difference, *μ* = 0).

The mean and standard deviation of HSV values, overall surface irregularity. and maximum surface irregularity were calculated as descriptive statistics for these parameters. The mean and standard deviation of the interobserver differences for color values and irregularity measures were computed as a measure of precision and variation of color and irregularity acquisition of the intraoral scan. A *p* value < 0.05 was considered statistically significant.

## Results

Ten volunteers (five male and five female) were recruited; all subjects met the inclusion criteria.

### Trueness, precision, and scan time of the 3D palatal soft tissue models

**Fig. 3:**
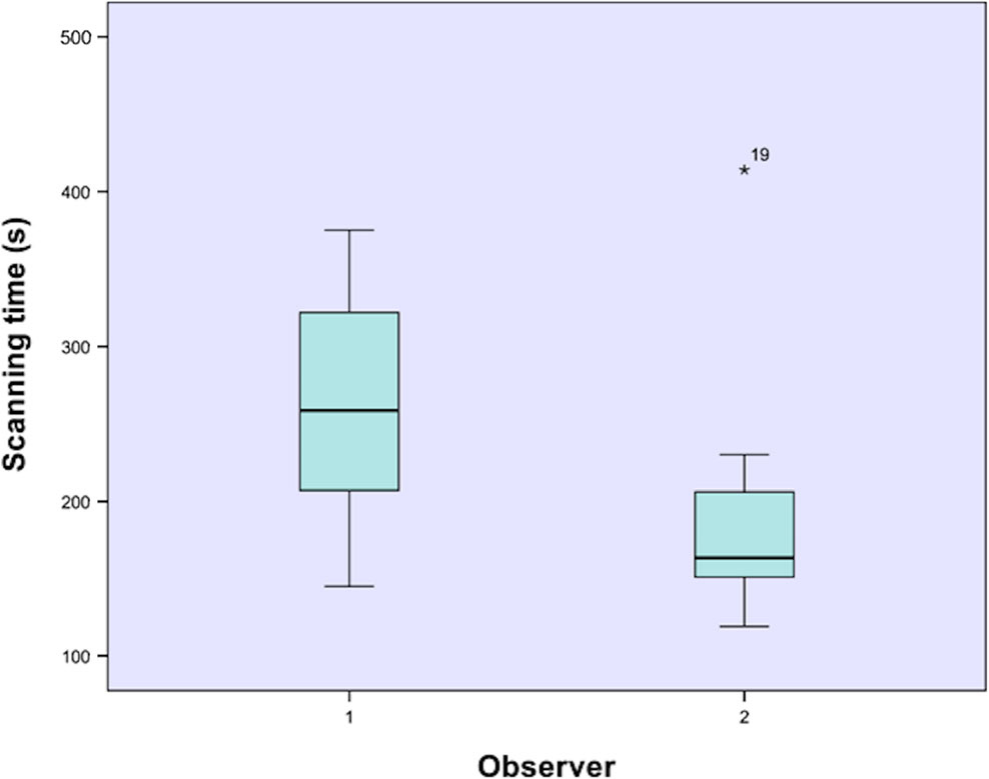
Box plot demonstrating scan time for palatal soft tissues and full upper dentitions of both observers. There was no statistical significance (*p* = 0.15)

Descriptive statistics for the absolute average and 90th percentile distances (absolute average distance, absolute 90th percentile distance) between IOS models and digitized impressions (trueness), as well as for the absolute average distance and absolute 90th percentile distance between the different IOS models (precision), are provided in Table 1. The mean absolute distance error (size measurement) between the digitized impression and the IOS model was 0.02 ± 0.07 mm which was statistically not significant (*p* = 0.30). The mean scan time for observer 1 was 262 ± 84 s; the mean scan time for observer 2 was 193 ± 76 s) (Fig. 3). This difference was statistically not significant (*p* = 0.15).

**Table 1:** Absolute inter-surface distances of the palatal area of both the conventional impression and the IOS of observer 1 and observer 2

	Absolute average distance (SD)	Absolute 90th percentile distance (SD)
IOS observer 1 and plaster cast (mm)	0.12 (0.02)	0.23 (0.05)
IOS observer 2 and plaster cast (mm)	0.14 (0.03)	0.28 (0.07)
IOS observer 1 and IOS observer 2 (mm)	0.08 (0.03)	0.16 (0.07)

### Precision of color

**Fig. 4:**
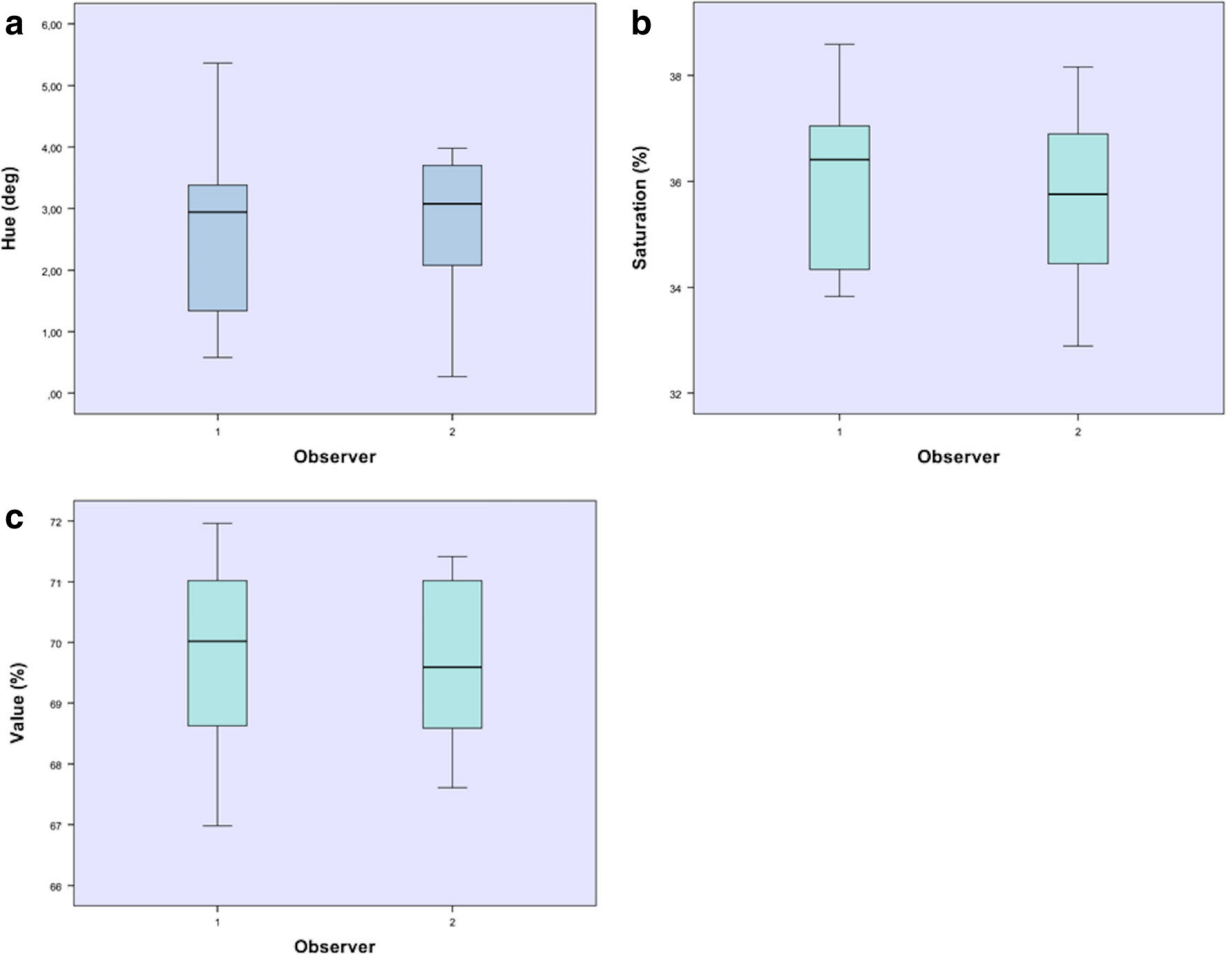
**a**, **b**, **c** Box plot showing the interobserver variability for hue (**a**), saturation (**b**), and value (**c**)

The results of the palatal color measurements are depicted in Table 2. The mean color of the palate for both observers in the HSV color scale was hue 2.69 ± 1.32°, saturation 35.86 ± 1.65%, and value 69.76 ± 1.42%. The mean interobserver difference of the color measurements was H − 0.08 ± 1.49, S 0.28 ± 0.78, V 0.30 ± 1.14 (Fig. 4).

**Table 2:** The results of the palatal color measurements

	Mean color observer 1 (SD)	Mean color observer 2 (SD)	Mean interobserver difference (SD)	*P* value
Hue (°)	2.65 (1.47)	2.73 (1.24)	− 0.08 (1.49)	0.864
Saturation (%)	36.00 (1.66)	35.72 (1.72)	0.28 (0.78)	0.286
Value (%)	69.91 (1.56)	69.61 (1.32)	0.30 (1.14)	0.426

There are no significant differences between the two observers measured

### Precision of surface irregularity

**Fig. 5:**
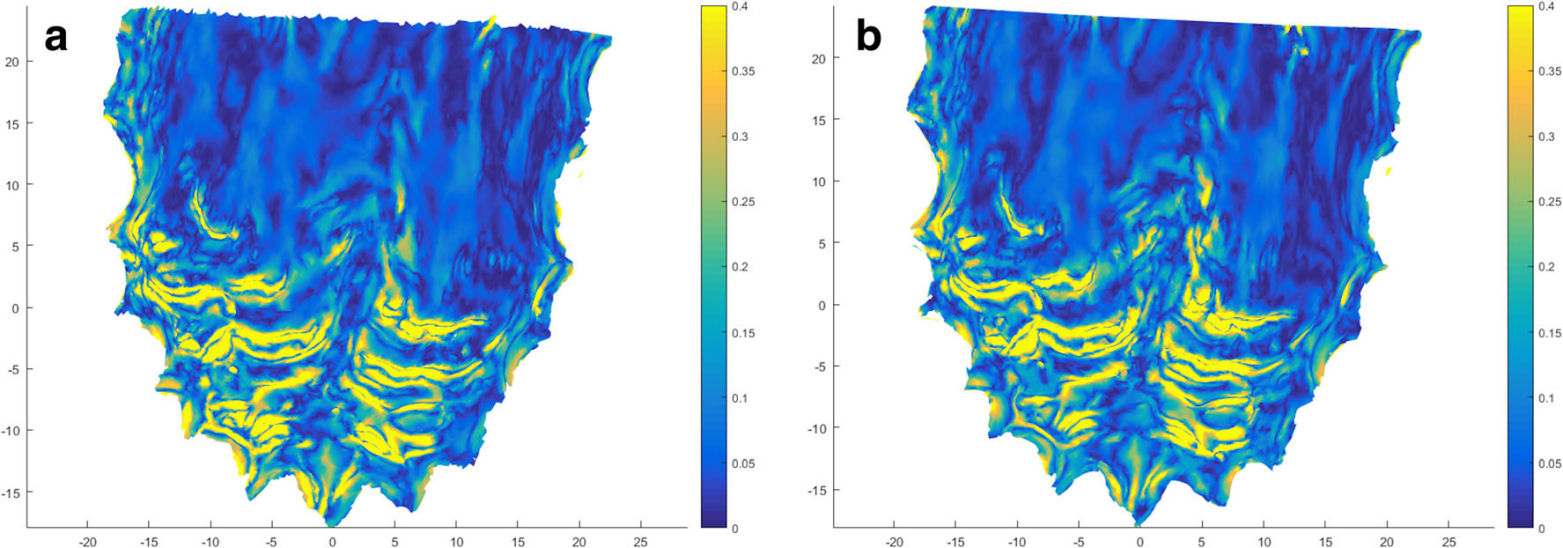
Heat map visualizing areas with strong (yellow) and weak (blue) curvature of the palate. Measurements of observer 1 (**a**) and observer 2 (**b**) are demonstrated

The mean overall surface irregularity was 0.16 ± 0.03. The maximum surface irregularity was 0.38 ± 0.07 mm. The mean interobserver differences for overall and maximum surface irregularity were 0.01 ± 0.03 and 0.00 ± 0.05 mm, respectively. A color-coded irregularity map of a volunteer’s palate was provided for observers 1 and 2 (Fig. 5).

## Discussion

To date, there is a lack of valuable, objective, and reproducible documentation of oral soft tissue lesions. All clinicians have different experience and expertise; a variety of measurement protocols are described and written reports with different descriptive vocabulary exist. Epstein et al. [2] conducted a systematic review to assess the effectiveness of the COE in predicting histological diagnosis of dysplasia or oral squamous cell cancer (OSCC). The authors found a high sensitivity of COE (0.93), but on the other hand a poor specificity (0.31). The results showed that COE alone may not detect OSCC and may not reliably discriminate between OSCC and benign, dysplastic lesions, since OSCC may manifest in mucosa that appears to be clinically normal [2, 9, 10]. Two-dimensional photographs can be obtained to complement the oral documentation; however, it is very hard to generate these pictures in a uniform style. These elements make adequate soft tissue analysis and comparison in terms of size, color, and surface texture infeasible. New adjunctive diagnostic aids like toluidine blue staining (TBlue), a vital dye that may stain nucleic acids and abnormal tissues, light-based systems as tissue reflectance (ViziLite™, Zila Pharmaceuticals; MicroLux™ DL, AdDent, Danbury, Connecticut), and narrow-emission tissue fluorescence (VELscope™, LED Medical Diagnostic Inc., Barnaby, Canada) are developed to enhance the detection of potentially malignant lesions. Although those techniques or technologies may play a major role in screening and diagnosing oral cancer, there is no clearly defined evidence to suggest that they improve the sensitivity, specificity, and positive predictive value beyond COE alone [4]. Moreover, the result of these adjunctive tests is a “live snapshot” of the imaged region, which makes follow-up and comparison infeasible and interpretation still dependent on the experience of the practitioner.

Nowadays, 3D digital intraoral scanning is gaining popularity in various fields of dentistry, including prosthodontics, implant dentistry, orthodontics, and oral and maxillofacial surgery. It has proven to be a valuable and accurate digital impression tool [6–8]. Intraoral scanning offers different advantages: no potential deformation (expansion, shrinkage, distortion) of the impression, more patient comfort, limited risk of spreading infections, and decreased working time [11]. Several authors investigated the trueness of the IOS using the conventional impression methods as a gold standard. Accurate results were described when focusing on single tooth preparations or small areas of the dental arch [12–15]. However, results were less congruent when the full dental arch was scanned. Patzelt et al. [16] and Zhang et al. [7] used different types of intraoral scanning devices to investigate trueness and precision values in respectively the edentulous jaw and full-arch dentition jaw. Patzelt et al. do not recommend IOS for digitization of the edentulous jaw, in contrast to Zhang et al., who suggested that IOS could be accepted as a clinical alternative of plaster models. Ender et al. [6] concluded that digital impressions were equal to or better than conventional impressions with irreversible hydrocolloid or polyether, but highly accurate conventional impression materials (Vinyl Siloxane Ether) were superior to the intraoral scan in terms of precision.

Gan et al. [8] performed a digital intraoral scan of the full upper jaw with the TRIOS® 3D scan. To assess trueness, a surface-based registration between the digitized impression and a plaster cast obtained with a conventional impression material was compared. Precision was studied by comparing repeated intraoral scans. Different models were aligned by utilization of a best fit algorithm. They reported an excellent precision on dental and palatal level (59.52 ± 11.29 vs 55.26 ± 11.21 μm, respectively). However, trueness on the palatal level significantly differed from the dental level (130.54 ± 33.95 vs 80.01 ± 17.78 μm, *p* < 0.05). Lower levels of trueness between IOS and conventional impression could be explained by the flexibility of the palatal mucosa. The intraoral scan has no touch with oral tissue, but a conventional impression is pressed against oral structures, possibly causing deformation. The present study reports lower results of precision on the palatal level (80 μm vs 55.66 μm). This could be attributed to the different method of model alignment in both studies (best fit on dental vs palatal level). The authors of this in vivo study aligned the models on dental level to assess an objective visualization of the palatal soft tissue. This method enables the possibility to evaluate soft tissue changes at different scanning moments.

To check the intraoral scan trueness, the authors used an irreversible hydrocolloid impression material as a gold standard. This material is routinely used in daily practice but is known to be less resistant to possible deformation compared to highly accurate materials as vinyl polysiloxane materials. To avoid any additional distortions when manufacturing the gypsum cast, Gan et al. [8] scanned the impressions immediately with the optical scanner since earlier studies mentioned that shadowing effects in the impression might have had a negative impact on data acquisition of cavities or negative molds [17]. This might have affected the integrity of the final virtual model in our study. Nonetheless, when comparing with Gan et al. [8], the current study found similar results of trueness on a palatal level (observer 1120 μm and observer 2140 μm vs 130.54 μm). These results confirm the possibility to make an objective digital image of the palatal surface and support the feasibility to use an optical scan of the conventional impression as a reference model.

The mean scanning time in this study (4 min 18 s ± 1 min 39 s) was also comparable with the results (4 min 58 s ± 1 min 17 s) of Gan et al. [8] where the same procedure (scanning of the upper jaw including the palate) was performed with the same equipment (TRIOS® 3D scan). The difference in scanning time between the trained observer (3 min 22 s ± 1 min 33 s) and the inexperienced investigator (4 min 37 s ± 1 min 21 s) emphasize the importance of training the scanning procedure. Park et al. [18] showed that appropriate training could change the efficiency of intraoral scan positively. In the current study, the longer scan time did not have an influence on trueness of the palatal tissue. However, it could be clinically relevant if more motile regions (e.g., tongue) need to be scanned. As the scanner captures single pictures that are stitched together into a three-dimensional network, an image mismatch may be more likely with increased scanning time in a complex or moving object.

To the best of our knowledge, this is the first study which attempts to use an intraoral scan in documentation of the oral mucosal surface in terms of color and surface irregularity. The possibility to define these additional objective clinical features is helpful to render a realistic image of an intraoral lesion and may play a crucial role to recognize abnormal lesions in early stage and record any changes over time. The TRIOS® 3D scanner can acquire lifelike colors in detail [5]. There is a broad color spectrum of intraoral lesions ranging from white or red to blue, brown, black, or mixed pigmentation. The interobserver variability of the palatal soft tissues showed almost perfect agreement for hue (−0.08 ± 1.49°) and for saturation (0.28 ± 0.78%). More variable results in terms of value (0.30 ± 1.14%) were measured. The reason for the variability of brightness could be attributed to different illumination angles. This study proved the feasibility to examine the average color of the complete palatal area. However, future investigations should assess the possibility to determinate the average color of a defined, smaller intraoral area. This would enable comparison of changes in color of mucosal abnormalities.

Another important clinical feature of potential oral malignancy is the surface texture of the lesion. Until now, this could only be described in terms of flat, elevated, or depressed, based on the palpation of the clinical practitioner [1, 19]. The irregularity values found between the different scan observers was highly reproducible (overall and maximum surface irregularity were 0.01 and 0.00 mm, respectively).

With objective parameters in terms of size, color, and surface roughness, the standard clinical examination would be enhanced. In this way, the clinician could offer a standard follow-up of suspected lesions and detect any changes in time. Moreover, the intraoral scanner could facilitate multidisciplinary consultations with reliable exchange of clinical information.

Nevertheless, this study has some limits. Since this was a validation study of a new technique, we examined a homogenous, fully dentate population without oral pathology. To assess the feasibility of our procedure, we scanned the palatal surface, as this is fixed, relatively dry intraoral mucosa with easy access. More difficulties can be expected when implementing this technique in routine clinical practice: patients with limited jaw opening or excessive saliva production or when mobile mucosal parts are scanned (e.g., tongue, mouth floor, cheek). Higher difficulty is expected when scanning an edentulous jaw, since we have only a few reference points and matching on dentition is not possible. Although IOS of the palatal mucosa is promising, more obstacles should be tackled before clinical implementation of this technique.

## Conclusion

It can be concluded that the application of the IOS for 3D documentation of palatal soft tissue in terms of shape, color, and curvature is likely as an adjunctive tool to COE. By validation of the proposed study, new clinical applications of the intraoral scanner can be examined in the future.
